# Low expression of ferritinophagy-related NCOA4 gene in relation to unfavorable outcome and defective immune cells infiltration in clear cell renal carcinoma

**DOI:** 10.1186/s12885-020-07726-z

**Published:** 2021-01-05

**Authors:** Yanhua Mou, Jinchun Wu, Yao Zhang, Omar Abdihamid, Chaojun Duan, Bin Li

**Affiliations:** 1 Department of Oncology, Xiangya Hospital, Central South University, Changsha, Hunan 410008 P.R. China; 2 Department of Oncology, Hubei Cancer Hospital, Affiliated Hubei Cancer Hospital of Huazhong University of Science and Technology, Wuhan, 430079 P.R. China; 3 Institute of Medical Sciences, Key Laboratory of Cancer Proteomics of Chinese Ministry of Health, Xiangya Hospital, Central South University, Changsha, Hunan 410008 P.R. China; 4 State Key Laboratory of Reproductive Medicine and Department of Urology, The First Affiliated Hospital of Nanjing Medical University, Nanjing, Jiangsu 210029 P.R. China; 5 Department of Thoracic Surgery, Xiangya Hospital, Central South University, Changsha, Hunan 410008 P.R. China; 6 National Clinical Research Center for Geriatric Disorders, Xiangya Hospital, Central South University, Changsha, Hunan 410008 P.R. China

**Keywords:** Ferritinophagy, NCOA4, Clear cell renal carcinoma, Immune cells, Ferroptosis

## Abstract

**Background:**

Clear cell renal cell carcinoma is susceptible to ferroptosis, and immunotherapy is recently recommended as a priority for the initial treatment of metastatic clear cell renal carcinoma. Increased ferroptosis and immune activation can synergistically reinforce each other in killing cancer cells. NCOA4 depletion can eliminate iron accumulation and thus weaken ferroptosis. Here, we aim to identify and validate the association between NCOA4 expression, clinicopathologic characteristics, and overall survival in ccRCC by using The Cancer Genome Atlas and Gene Expression Omnibus databases. We further analyze the interacted proteins of NCOA4 and infiltrated immune cells via TIMER and GEPIA databases.

**Methods:**

NCOA4 expression in clear cell renal carcinoma (ccRCC) tissues and normal adjacent tissues in The Cancer Genome Atlas (TCGA) data were primarily screened, and further validated in another independent cohort from the gene expression omnibus (GEO) database and human protein atlas. The relationships of NCOA4 expression and clinicopathologic parameters and overall survival (OS) were assessed using multivariate methods and Kaplan-Meier survival curves. And the proteins network with which NCOA4 interacted were also built using the online STRING website. Meanwhile, we use TIMER and GEPIA databases to investigate the relationships between NCOA4 expression and infiltrated immune cells and their corresponding gene marker sets.

**Results:**

Contrast to normal tissue, NCOA4 expression was lower in ccRCC tumor tissue(*p* < 0.05). Lower NCOA4 expression was closely associated with high-grade malignancy and advanced TNM stage. Univariate and multivariate analysis indicated the overall survival of ccRCC cases with low NCOA4 level is shorter than those of patients with high NCOA4 expression (*p* < 0.05). FTL and FTH1 were the important proteins interacting with NCOA4. ccRCC with NCOA4 deficiency presented the paucity of infiltrated immune cells and their matching marker sets, including CD8+ T cells.

**Conclusion:**

Deficient NCOA4 expression was related to disease progression and poor prognosis, as well as impaired infiltration of immune cells in ccRCC.

## Background

Over the past decades, the global incidence of renal cell carcinoma (RCC) is increasing which ranks first mortality among annual urologic cancers [1]. RCC is a heterogeneous cancer, most of them is clear cell RCC (ccRCC) which nearly accounts for 75–80% of RCC [2]. Surgery is the mainstay of ccRCC, which still present a high recurrence rate of 40% after radical surgery and have high mortality rate once it metastasizes to distant organs [1, 2].

Targeted therapies are currently one of the standard treatments for ccRCC, but almost all patients will ultimately develop disease deterioration because drug-induced cell apoptosis or autophage was escaped by ccRCC cells [3]. Therefore, as a novel cell death, ferroptosis-induction is becoming an alternative choice for the therapeutic strategy of ccRCC [4–7].

NCOA4, also named as androgen receptor-associated protein 70 (ARA70), was originally described as a coactivator of multiple nuclear hormone receptors. It was closely related to tumorigenesis and progression of ovarian cancer, prostate cancer, breast cancer and pancreatic cancer [8]. Recent studies unravel that NCOA4 is an autophagosomes component that participates in the process of ferritinophagy [8, 9]. Surface arginine in ferritin heavy chain 1 (FTH1) can specifically bind the C-terminal element of NCOA4 and fused with a lysosome via nascent autophagosomes, thus facilitating ferroptotic cell death [10]. NCOA4 was gradually considered as a key molecule promoting ferroptosis in various cancer cells and mounting studies displayed that NCOA4 depletion can inhibit ferroptosis by eliminating the accumulation of intracellular free iron, glutathione production and reactive oxygen species (ROS) [11].

Immunotherapy is coming into clinical practice in the treatment of ccRCC due to FDA approval and NCCN recommendation [12]. Recently, two independent studies simultaneously reported the same finding that ferroptosis provoked by T cells in cancer cells is an important anti-tumor method of PD-1/PD-L1 antibody and weak effect of PD-L1 antibody was observed in the ferroptosis-insensitive tumor cells [13, 14]. Increased ferroptosis can also advance the anti-tumor effect of immunotherapy which pinpointed the positive feedback between ferroptosis and immunotherapy, cooperatively killing cancer cells [13].

In our study, we downloaded and analyzed the relationship of NCOA4 expression with clinical information and overall survival in ccRCC patients utilizing various databases of TCGA, GEO and human protein atlas. And then we used TIMER and GEPIA databases to investigate the relationships between NCOA4 expression and infiltrated immune cells and their corresponding gene marker sets. Also, the NCOA4-interacted protein network was explored utilizing the STRING website. The results showed that low NCOA4 level acted as an indicator for poor prognosis and was associated with deficient infiltration of immune cells in ccRCC. Thus, it is plausible that NCOA4 defect reduce ferroptosis and thus possibly debilitate antitumor immune effects in ccRCC. Targeting NCOA4 may be a promising therapeutic strategy for ferroptosis-induction or/and with the combination of immunotherapy in ccRCC.

## Methods

### Data source

The Cancer Genome Atlas (TCGA) (https://genome-cancer.ucsc.edu/), a free data portal of largescale cancer genome project, provides clinic and pathological information of 33 types of cancer for scholars and researchers. The data of ccRCC patients with the expression of RNA-Seq and matching clinical pathologic information were obtained by the TCGA tools cancer browser. The database is publicly open-access and available and therefore there was no need to get approval from the local ethics committee.

### The GEO database and the human protein atlas

As one of the biggest collection of gene chips in the world, the GEO database is a comprehensive gene expression library in the National Center of Biotechnology Information (NCBI) (https://www.ncbi.nlm.nih.gov/geo/). The Human Protein Atlas offers a broad amount of proteomic and transcriptome information of distinct human samples, which consists of cell, tissue and pathology Atlas. To date, the online database provides cell-specific location information for 44 various normal tissues and 20 of the most typical categories of cancer. Moreover, protein immunohistochemistry in normal human tissues and tumor tissues can also be obtained from this online website.

### Survival and statistical analysis

According to the median expression of NCOA4 gene, patients in test and validation set were split into two groups of high NCOA4 expression and low NCOA4 expression respectively. To investigate whether NCOA4 expression level affects the clinical outcomes of ccRCC patients, we constructed a prognostic classifier using Kaplan-Meier (KM) survival curves to compare the survival disparities.

### Univariate and multivariate logistic regression analysis

To further determine the effect of NCOA4 expression in ccRCC patients, we use univariate Cox regression analysis for calculating the association between the expression level of NCOA4 and patient’s OS in two cohorts. Afterwards, a multivariate analysis was used to assess if the NCOA4 is an independent prognostic factor for ccRCC patient survival. The NCOA4 has statistical significance in Cox regression analysis when *p* value is less than 0.05.

### Protein-protein interaction comprehensive analysis

Another online tool we used was the Search Tool for the Retrieval of Interacting Genes/Proteins (STRING) website (https://string-db.org/). The website hosts a big collection of integrated and consolidated protein-protein interaction data. After importing the NCOA4 into the online tool STRING, we obtained the protein–protein interaction (PPI) network information. The confidence score > 0.7 was considered significant.

### TIMER database analysis

The Tumor Immune Estimation Resource (TIMER) is a public website which covers 32 cancer types and encompasses 10,897 samples from TCGA database, aiming to assess the abundance of immune inner infiltrates (http://cistrome.org/TIMER/). The correlation of NCOA4 expression with the abundance of six types of infiltrating immune cells (CD8+ T cells, CD4+ T cells, B cells, dendritic cells macrophages, and neutrophils) in ccRCC patients was evaluated via TIMER database. The relationship between the expression of the NCOA4 gene and the tumor purity was also displayed.

### Gene correlation analysis

The Gene Expression Profiling Interactive Analysis (GEPIA) (http://gepia.cancer-pku.cn/index.html) is an online database that consists of 9736 tumors and 8587 normal samples from TCGA and GTEx data. It focuses on the analyses of the expression of RNA sequencing. Gene Classes and Isoform Classes exhibit the types of 60,498 genes and 198,619 isoforms. In the GEPIA database, the relation of NCOA4 expression with multiple markers for immune cells was investigated. The x-axis was presented with the level of NCOA4 expression, and the y-axis was plotted with other interest genes. In addition, we used TIMER data to validate the genes which were of significant correlation with NCOA expression in the GEPIA web.

## Results

### Patient characteristics

In total, the RNA-sequencing data and detailed clinical prognostic information resources of 533 ccRCC samples and 72 normal tissue samples from the TCGA database were incorporated into our research. All patients were randomly grouped into test set (*n* = 355) and validation set (*n* = 178). We summarized the clinical information including age at diagnosis, gender, laterality, histologic grade, pathologic stage (T, N or M), OS time and survival outcomes in Table 1.

**Table 1:** Clinical characteristics of the ccRCC patients in test and validation sets

Clinical factor	Test set	Validation set	Overall
	(*n* = 355)	(*n* = 178)	(*n* = 533)
**Age**			
Mean	60.73802817	60.40449438	60.62664165
Median[min, max]	61[26, 88]	60[29, 90]	61[26, 90)
**Gender**			
Male	235(66.2%)	68(38.2%)	303(56.8%)
Female	120(33.8%)	110(61.8%)	230(43.2%)
**Overall Survival time**			
Mean	1401.886686	1243.398876	1348.758945
Median[min, max]	1230[3, 4537]	1131.5[2, 3987]	1191[2, 4537]
Missing	2	0	2
**Survival State**			
Living	240(67.6%)	116(65.2%)	356(66.8%)
Dead	113(31.8%)	62(34.8%)	175(32.8%)
Missing	2(0.6%)	0(0%)	2(0.4%)
**Laterality**			
Left	170(47.9%)	81(45.5%)	251(47.1%)
Right	184(51.8%)	97(54.5%)	281(52.7%)
Missing	1(0.3%)	0(0%)	1(0.2%)
**Histologic grade**			
G1	10(2.8%)	4(2.2%)	14(2.6%)
G2	152(42.8%)	77(43.3%)	229(43.0%)
G3	139(39.2%)	67(37.6%)	206(38.6%)
G4	51(14.4%)	25(14.1%)	76(14.3%)
Missing	3(0.8%)	5(2.8%)	8(1.5%)
**Pathologic T**			
T1	180(50.7%)	93(52.3%)	273(51.2%)
T2	51(14.4%)	18(10.1%)	69(12.9%)
T3	118(33.2%)	62(34.8%)	180(33.8%)
T4	6(1.7%)	5(2.8%)	11(2.1%)
**Pathologic N**			
N0	164(46.2%)	76(42.7%)	240(45.0%)
N1	11(3.1%)	5(2.8%)	16(3.0%)
Missing	180(50.7%)	97(54.5%)	277(52.0%)
**Pathologic M**			
M0	284(80.0%)	138(77.5%)	422(79.2%)
M1	48(13.5%)	31(17.4%)	79(14.8%)
Missing	23(6.5%)	9(5.1%)	32(6.0%)
**Pathologic stage**			
I	175(49.3%)	92(51.7%)	267(50.1%)
II	45(12.7%)	12(6.7%)	57(10.7%)
III	83(23.4%)	40(22.5%)	123(23.1%)
IV	50(14.1%)	34(19.1%)	84(15.7%)
Missing	2(0.5%)	0(0%)	2(0.4%)

### Lower NCOA4 expression in tumor samples than that in normal tissues

**Fig. 1:**
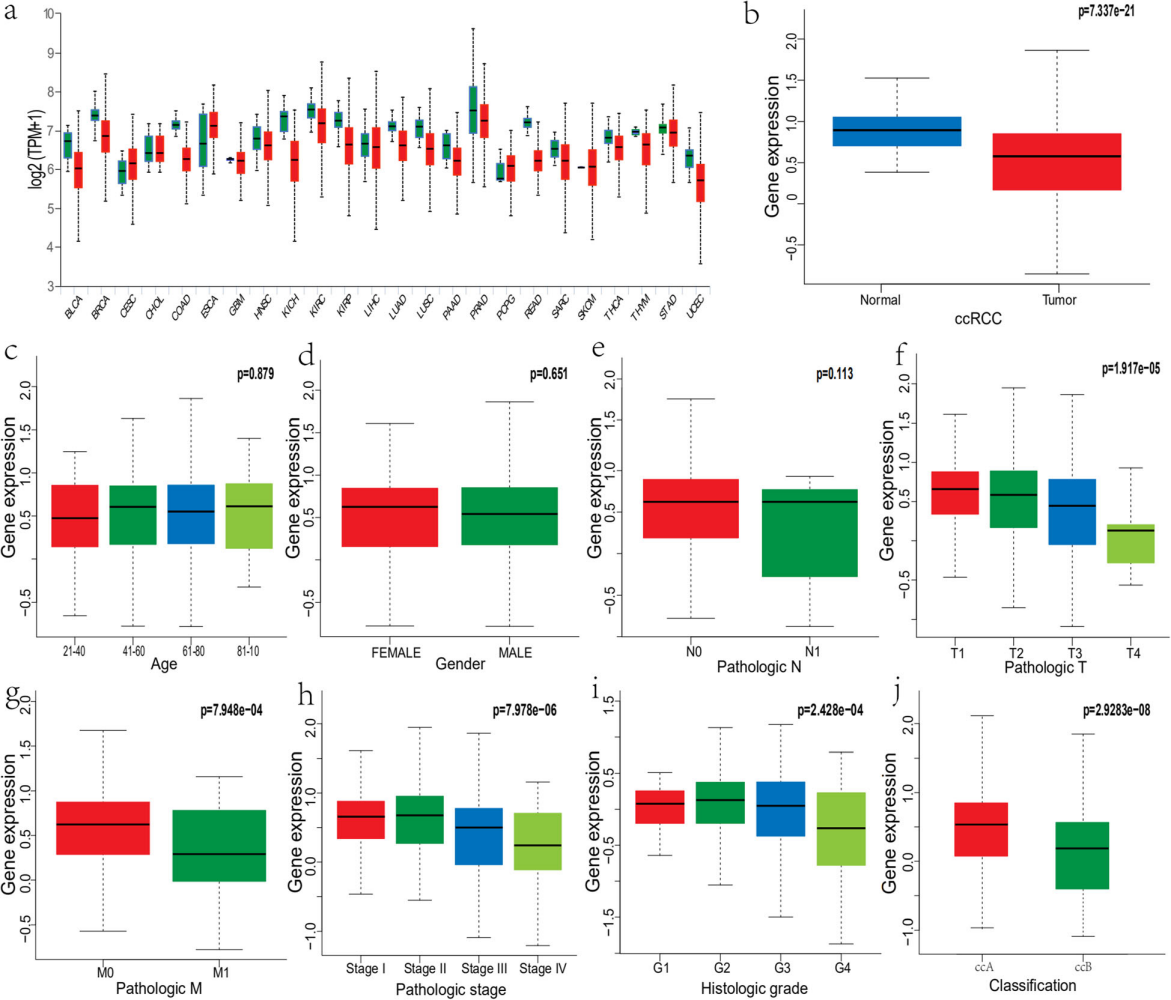
NCOA4 expression status in cancers. **a** Human NCOA4 expression levels in different cancer tissues and corresponding normal tissues. **b** Compared with normal tissues, the expression levels of NCOA4 was significantly decreased in ccRCC tissues. **c**-**e** There was no statistically significant difference between NCOA4 mRNA levels and age, gender and pathologic N stage. **f**-**j** Lower NCOA4 expression was associated with higher pathologic T, M, and tumor stage as well as higher grade and ccRCC classification (*p* = 1.917e-05) (*p* = 7.948e-04) (*p* = 7.978e-06) (*p* = 2.428e-04) (*p* = 2.9283e-08)

**Fig. 2:**
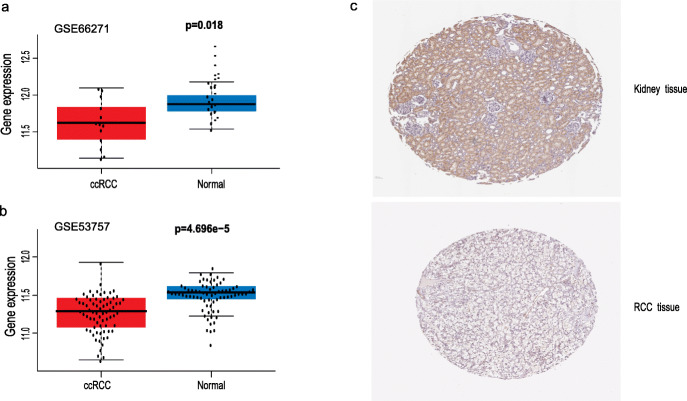
Analysis of the NCOA4 gene expression in GEO datasets and the human Protein atlas. **a** Validation of lower NCOA4 mRNA expression in ccRCC than that in normal tissue in GSE66271 dataset. **b** Validation of lower NCOA4 mRNA expression in ccRCC than that in normal tissue in GSE53757 dataset. **c** The level of NCOA4 protein in RCC tissue was lower than that in normal tissue in the Human Protein Atlas (Antibody HPA065208, 10X)

The mRNA expression level of NCOA4 was analyzed in various cancer types. The gene expression level of NCOA4 was significantly lower in tumor samples in comparison to normal tissues of ccRCC in TCGA database(*p* = 7.337e-2) (Fig. 1), which was also validated in GEO database (*p* = 4.696e-05, 0.018) (Fig. 2). Correspondingly, the expression of NCOA4 protein is downregulated in ccRCC tissue as compared to normal tissue in comparison to that in normal tissue in the Human Protein Atlas. In the analysis of the correlation of NCOA4 expression and clinicopathologic parameters in ccRCC patients, the results show that no significant difference between NCOA4 mRNA levels and age (*p* = 0.879), gender (*p* = 0.651) and pathologic N stage (*p* = 0.113). But lower NCOA4 expression level was observed in higher T stage and M stage and tumor stage (*p* = 1.917e-05, 7.948e-04, 7.978e-06), as well as in higher grade and ccRCC classification (*p* = 2.428e-04, 2.9283e-08).

### Lower NCOA4 mRNA expression showing shorter OS in ccRCC

**Fig. 3:**
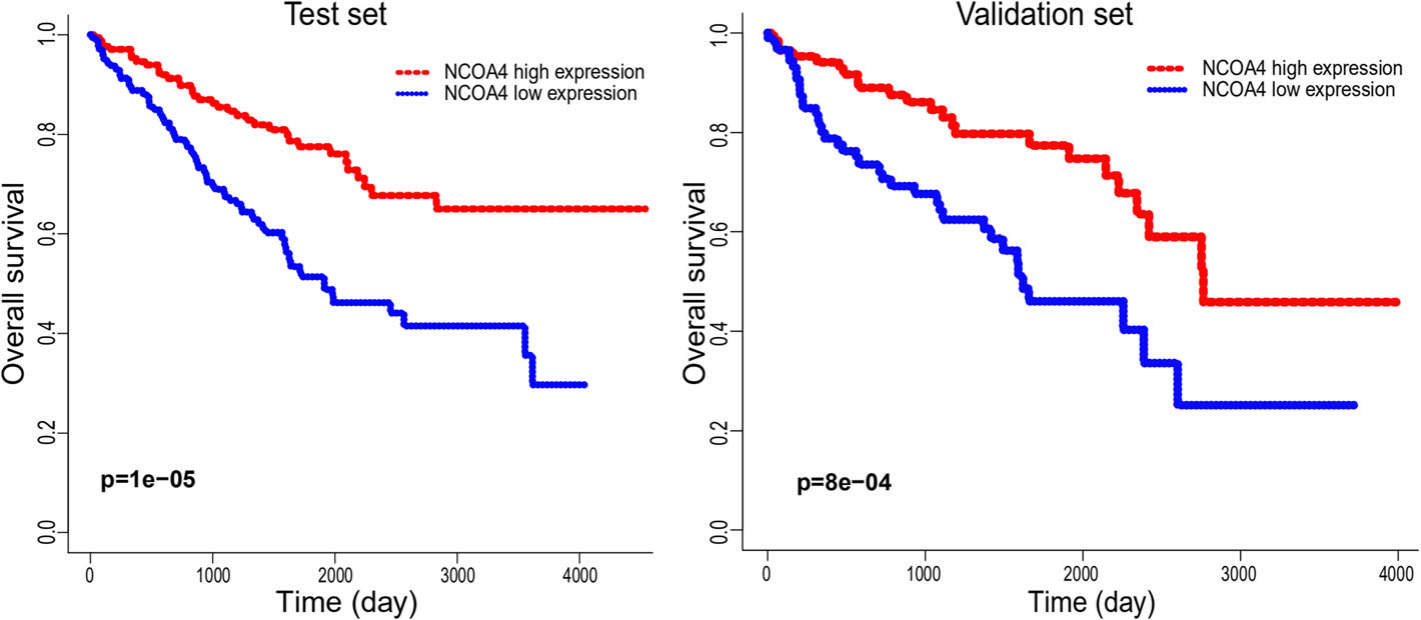
The Kaplan-Meier survival curves of the ccRCC patients with high and low NCOA4 expression level

**Fig. 4:**
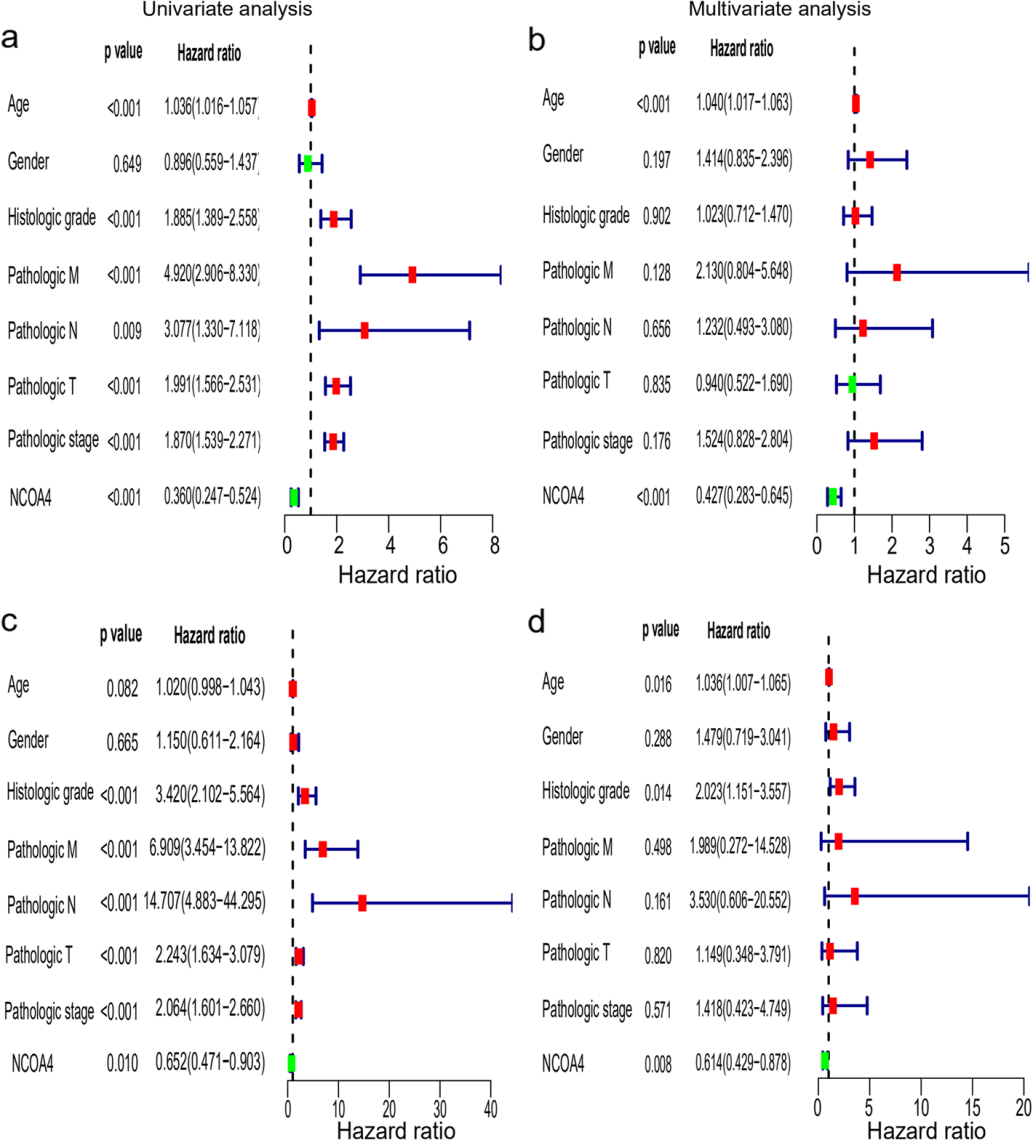
Univariate and multivariate regression analysis of NCOA4 and other clinicopathologic parameters with OS in ccRCC patients

According to the KM plots, ccRCC cases with lower NCOA4 mRNA expression have shown a shorter overall survival (OS) in the test cohort (*p* = 1e-5), and was also be validated in an independent ccRCC cohort (*p* = 8e-4) (Fig. 3). In the univariate Cox model, both low NCOA4 expression and high pathologic grade and stage (TNM) were a negative predictor for OS in ccRCC patients which was confirmed in the test set and validation set. Intriguingly, in multivariate regression analysis, NCOA4 expression was independent factor correlated with OS both in the test set (*p* < 0.01) and validation set (*p* = 0.008) (Fig. 4).

### Constructing protein interaction networks

**Fig. 5:**
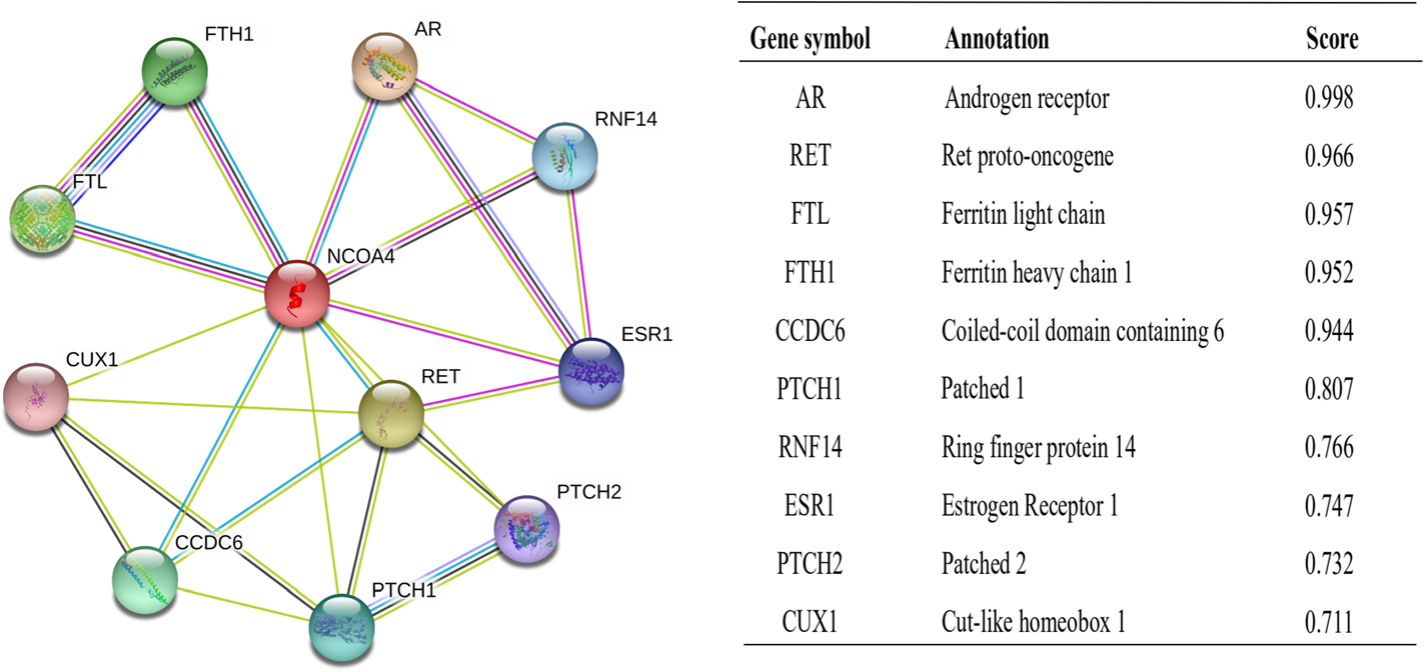
NCOA4-interaction proteins in ccRCC tissue. Annotation of NCOA4-interacting proteins and their co-expression scores

The functional interaction between proteins is necessary for the molecular mechanism and metabolism of malignancy. Therefore, we used STRING tool to analyze the PPI network of NCOA4 protein to determine their interactions in the progression of ccRCC. The top 10 proteins and corresponding gene names, annotations and scores are listed in Fig. 5. These genes included: AR, RET, FTL, FTH1, CCDC6, PTCH1, PNF14, ESR1, PTCH2, CUX1. The RET fusion is becoming a potential novel target in solid tumors [15]. FTL and FTH11 are the main factors that regulate iron metabolism. Elevated FTH1 mRNA levels were correlated with worse prognosis of RCC patients [16].

### Correlation analysis between NCOA4 expression and infiltrating immune cells

**Fig. 6:**
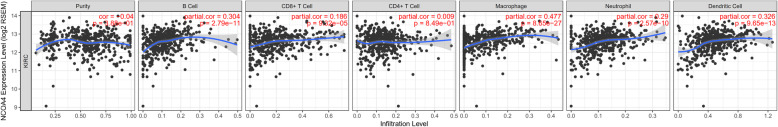
Correlation of NCOA4 expression with infiltrating immune infiltration in ccRCC

Tumor infiltrating lymphocytes affect the survival of patients with various cancers. Therefore, we analyzed the correlation of NCOA4 expression with six kinds of infiltrating immune cells (CD8+ T cells, CD4+ T cells, B cells, dendritic cells macrophages, and neutrophils) and tumor purity. The results displayed that the expression level of NCOA4 had obviously positive correlation with infiltrating levels of B cells (*r* = 0.304, *P* = 2.79e-11), CD8+ T cells (*r* = 0.186, *P* = 9.32e-05), macrophages (*r* = 0.477, *P* = 8.65e-27), neutrophils (*r* = 0.29, *P* = 2.57e-10), and dendritic cells (*r* = 0.326, *P* = 9.65e-13) in ccRCC, but no association with tumor purity and CD4+ T cells. *P* < 0.05 was considered as the difference is of significance (Fig. 6).

However, there was no significant correlation between NCOA4 expression with the amount of infiltrated CD4+ T cells and tumor purity in ccRCC. To intensely explore the possible role of NCOA4 in the infiltration of various immune cells in ccRCC, we used the GEPIA and TIMER databases to execute the relationships between NCOA4 and several immune marker sets, which were widely accepted as corresponding symbols of different immunocytes, such as CD8+ T cells, T cells (general), B cells, M1/M2 macrophages, tumor-associated macrophages, neutrophils, monocytes, NK, and DCs in ccRCC (Table 2). Furthermore, various functional T cells including Th1, Th2, Th9, Th17, Th22, Tfh, exhausted T cells, and Treg, were also be examined in our study. Results showed that the levels of most immune sets marking different T cells, TAMs, M1/M2 macrophages, monocytes and DCs were associated with the NCOA4 expression in ccRCC.

**Table 2:** Correlation analysis between NCOA4 and markers of immune cells in TIMER and GEPIA

Cell type	Gene marker	None		Purity		Tum our		Normal	
		Cor	*P*	Cor	*P*	R	*P*	R	*P*
B cell	CD19	−0.068	0.119	−0.069	0.139	−0.072	0.1	−0.16	0.18
	CD20(KRT20)	−0.023	0.593	−0.014	0.771	0.073	0.096	−0.087	0.47
	CD38	0.304	***	0.299	***	0.13	**	0.081	0.5
CD8+ T cell	CD8A	0.112	**	0.089	0.0567	0.11	*	−0.15	0.22
	CD8B	0.074	0.0897	0.046	0.329	0.09	*	−0.23	0.053
Tfh	BCL6	0.076	0.0807	0.066	0.159	0.17	***	0.13	0.27
	ICOS	Cor	***	0.203	***	0.19	***	−0.059	0.62
	CXCR5	−0.034	0.431	−0.022	0.634	−0.52	***	−0.29	*
Th1	T-bet (TBX21)	0.061	0.	0.04	0.393	0.08	0.067	−0.14	0.26
	STAT4	0.065	0.136	0.052	0.269	0.1	*	−0.016	0.89
	IL12RB2	0.241	***	0.216	***	0.25	***	0.093	0.44
	WSX1(IL27RA)	−0.069	0.113	−0.1	*	−0.034	0.43	0.072	0.55
	STAT1	0.484	***	0.476	***	0.42	***	0.26	*
	IFN-γ (IFNG)	0.027	0.531	0.005	0.917	0.043	0.33	−0.16	0.17
	TNF-α (TNF)	0.146	***	0.154	***	0.15	***	0.17	0.15
Th2	GATA3	−0.072	0.955	−0.042	0.364	−0.085	0.053	0.3	**
	CCR3	0.203	***	0.205	***	0.092	*	−0.033	0.78
	STAT6	0.377	***	0.367	***	0.3	***	0.22	0.069
	STAT5A	0.355	***	0.344	***	0.38	***	0.15	0.2
Th9	TGFBR2	0.62	***	0.593	***	0.59	***	−0.089	0.46
	IRF4	0.151	***	0.151	**	0.04	0.36	−0.12	0.3
	PU.1(SPI1)	0.063	0.144	0.047	0.318	0.083	0.058	−0.082	0.49
Th17	STAT3	0.599	***	0.592	***	0.6	***	0.33	**
	IL-21R	0.151	***	0.142	**	0.11	*	−0.19	0.11
	IL-23R	0.2	***	0.21	***	0.13	**	0.014	0.91
	IL-17A	−0.052	0.233	−0.026	0.581	−0.013	0.78	0.071	0.55
Th22	CCR10	−0.18	***	−0.185	***	−0.13	**	−0.18	0.14
	AHR	0.537	***	0.534	***	0.58	***	0.23	0.05
Treg	FOXP3	−0.082	0.0572	−0.096	*	−0.11	*	−0.05	0.68
	CD25(IL2RA)	0.312	***	0.293	***	0.1	*	−0.025	0.83
	CCR8	0.211	***	0.207	***	0.17	***	−0.099	0.41
T cell exhaustion	PD-1 (PDCD1)	−0.073	0.0939	−0.092	*	−0.011	0.8	−0.3	*
	CTLA4	−0.002	0.962	−0.005	0.911	0.017	0.69	−0.038	0.75
	LAG3	−0.074	0.0882	−0.088	0.0578	−0.022	0.61	0.14	0.23
	TIM-3 (HAVCR2)	0.366	***	0.337	***	0.31	***	−0.055	0.65
Macrophage	CD68	0.335	***	0.293	***	0.4	***	−0.049	0.68
	CD11b (ITGAM)	0.391	***	0.383	***	0.15	***	0.018	0.88
M1	INOS (NOS2)	0.349	***	0.324	***	0.13	**	0.0074	0.95
	IRF5	0.134	**	0.139	**	0.14	**	0.26	*
	COX2(PTGS2)	0.114	**	0.143	**	−0.019	0.66	0.26	*
M2	CD16	0.531	***	0.514	***	0.26	***	−0.029	0.81
	ARG1	0.069	0.111	0.046	0.32	−0.019	0.67	−0.21	0.079
	MRC1	0.583	***	0.567	***	0.51	***	0.13	0.28
	MS4A4A	0.461	0.0528	0.453	***	0.45	***	0.024	0.84
TAM	CCL2	0.084	***	0.114	*	0.075	***	0.033	0.78
	CD80	0.284	***	0.3	***	0.2	0.087	0.035	0.77
	CD86	0.389	***	0.392	***	0.44	***	0.065	0.59
	CCR5	0.301	***	0.3	***	0.35	***	0.16	0.17
Monocyte	CD14	0.154	***	0.132	**	0.16	***	0.032	0.79
	CD16(FCGR3B)	0.355	***	0.334	***	0.2	***	0.16	0.18
	CD115 (CSF1R)	0.387	**	0.376	***	0.36	***	0.039	0.74
Neutrophil	CD66b (CEACAM8)	0.122	***	0.12	*	0.078	***	0.29	*
	CD15(FUT4)	0.498	***	0.491	***	0.46	0.075	0.024	0.84
	CD11b (ITGAM)	0.391	0.0673	0.383	***	0.15	***	0.018	0.88
Natural killer cell	XCL1	−0.079	***	−0.086	0.0639	−0.039	***	−0.08	0.5
	CD7	−0.226	0.107	−0.271	***	−0.067	0.37	−0.26	*
	KIR3DL1	0.07	***	0.041	0.382	0.085	0.13	−0.17	0.15
Dendritic cell	CD1C(BDCA-1)	0.307	***	0.29	***	0.24	0.053	−0.026	0.83
	CD141(THBD)	0.26	*	0.243	***	0.29	***	−0.15	0.22
	CD11c (ITGAX)	0.1	***	0.115	*	0.034	***	−0.0063	0.96

*Tfh* Follicular helper T cell, *Th* T helper cell, *Treg* Regulatory T cell, *TAM* Tumor-associated macrophage. None, Correlation without adjustment. Purity, Correlation adjusted by purity. Cor, R value of Spearman’s correlation. *P < 0.05; **P < 0.01; ***P < 0.001

### Discussion

NCOA4 is a cargo receptor, which is specific for ferritin turnover by expediting ferritinophagy, thus it is crucial for iron homeostasis [9]. It promotes cell ferroptosis by degrading intracellular ferritin and causing iron retention, which indicates that NCOA4 is an important molecule in the process of ferroptosis in cancer [17]. NCOA4 depletion was reported to cause a cell disturbed ferroptosis process by eliminating the accumulation of intracellular free iron, glutathione and reactive oxygen species (ROS), and it was closely related to the tumorigenesis and progression of various cancers such as prostate cancer, ovarian cancer and breast cancer [8, 18].

ccRCC is the most common subtype of RCC [1]. More than 90% of ccRCC tumors show constitutive activation of the hypoxia-inducible factor (HIF) proteins resulting from biallelic inactivation of the tumor suppressor von Hippl-Lindau (VHL) gene, which underline the clear-cell phenotype of ccRCC because of abnormal lipid and glycogen accumulation, also accounting for its trait of resistance to chemotherapy and radiotherapy [19–22]. Recently, growing studies unraveled that ccRCC hold an innate susceptibility to ferroptosis because of its special metabolic states derived from the hypoxia-inducible factor pathway in ccRCC [6, 7].

Herein, we execute the study about the role of NCOA4 expression on tumorigenesis and progression, as well as the prognosis of ccRCC on the basis of various databases including TCGA, GEO and Human Protein Atlas. Expectedly, deficient NCOA4 was associated with the tumorigenesis and progression of ccRCC. ccRCC cases with lower NCOA4 expression showed inferior prognosis in comparison to that with higher NCOA4 expression. And correspondingly, as functionally distinct compositions of ferritin, FTL and FTH1 were also identified as the important proteins which interacted with NCOA4 molecule in ccRCC based on the analysis from STING software. Interestingly, sorafenib can induce cell ferroptosis of hepatic stellate cells (HSCs) by remarkably increasing NCOA4 expression, and thus improved the survival of the patients [23, 24]. These results indicated impaired ferroptosis resulting from NCOA4 deficiency may be the underlying mechanism for impaired NCOA4 as a negative predictor of ccRCC. Indeed, targeted ferritinophgic flux (NCOA4/ferritin) either by TGF-β1 or combined with DpdtpA has exhibited a remarkable antitumor effect [25, 26].

RCC has stood out as one of the most immune-infiltrated tumors, and clinically PD-1/PD-L1 antibody has been approved in the front-line setting of metastatic ccRCC [27]. However, although with convinced efficacy, some patients were still nonreactive to PD-1 antibody [28]. Recent study suggested that tumor infiltration lymphocytes with the state of T cell activation are strong prognostic determinants of ccRCC [29].

Our study demonstrated that the expression level of NCOA4 has a significantly consistent correlation with the infiltration levels of B cell, macrophages, dendritic cells and neutrophils in ccRCC. Further analysis of infiltrated lymphocyte markers showed that the markers of M1 macrophage such as NOS2, IRF5 and PTGS2 were weakly correlated with NCOA4 expression, whereas the gene markers of M2 macrophages such as MS4A4A, MRC1 and CD163 have a moderate relationship with NCOA level, which indicates the possible regulation role of NCOA4 in the polarization of TAM. Intriguingly, we found a strong correlation between NCOA4 and Tim-3, a vital marker gene of T-regulatory (Treg) cell exhaustion. Treg cells are notoriously known as the main manipulator creating immunosuppressive tumor microenvironment, and Treg cells infiltration within tumors were related to a higher pathological stage and poor prognosis of ccRCC [30, 31].

**Fig. 7:**
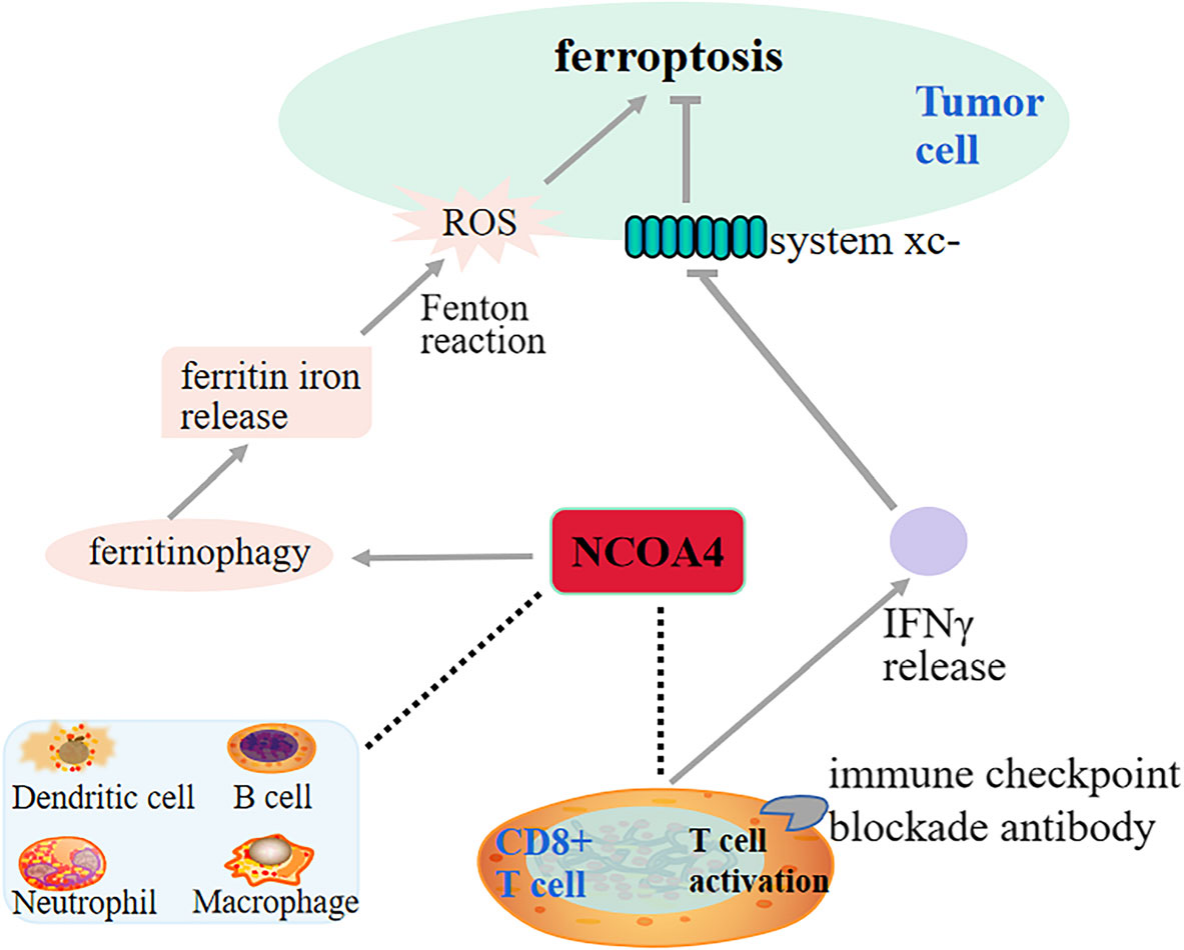
Overview of the relationship between NCOA4 and ferroptosis and immune checkpoint blockade

Compellingly, our finding unraveled that NCOA4 expression was strongly correlated with CD8+ T cells infiltration and its corresponding markers CD8A in ccRCC. CD8+ T cells are well-known effector cells of cancer immunotherapy [32, 33]. Traditionally, activated cytotoxic CD8+ T cells eliminate tumors mostly via irritating cell death in a Fas-Fas ligand pathways or by releasing perforin-granzyme [34, 35]. The latest two studies individually reported that CD8+ T cells activated by immunotherapy can induce ferroptosis by specifically enhancing lipid peroxidation in tumor cells, and that increased ferroptosis contributed to the antitumor efficacy of immunotherapy, further confirming the crucial role of ferroptosis in immunotherapy [13, 14]. Intriguingly, NCOA4 deficiency can impair the IFN-γ receptor signaling, which is a major effector of activated T cells for inducing ferroptosis in immunotherapy [36]. These results further implied that NCOA4 was the key molecule for bridging ferroptosis process and immunotherapy (Fig. 7).

Ferroptosis was gradually considered as a promising modality for developing effective combinational therapy strategies in the advancing era of cancer treatment [14, 37]. Due to the evolving first-line treatment choices in the patients with metastatic ccRCC, tumor biology, and tumor microenvironment should be considered upfront in predicting the optimum benefit from treatment strategies [26, 38, 39]. NCOA4 expression could be a potential novel factor for the stratification of ccRCC patients in guiding ferroptosis or/ and immunotherapy, which may be one of the main elements in a panel that reproducibly prognosticate the patients with ccRCC.

Notable limitations of our study include that lack of an already existing large quantity of tumor and normal samples analyses from the database, data heterogeneity, and platform differences. Our study shows that pathological grade and TNM stage are not independent prognostic factors for OS in patients with ccRCC, which is different from other studies [40]. This discrepancy may be due to data heterogeneity or the different grading and staging standards [41]. In addition, the group ethics information in the TCGA database is mainly limited to white and black populations, so it is hard to extrapolate these findings to other ethnicities. Prospective efforts focusing on the validation of the results drawn from the bioinformatics prediction, including proteins detection with a western blot or immunohistochemical staining and the functional analysis of NCOA4 in facilitating ferroptosis and immunotherapy in vivo and in vitro is further needed to advance the field.

## Conclusion

Taken together, ferroptosis induction and immunotherapy have been the major breakthroughs in ccRCC therapy. With the new progress in understanding the treatment biology and underlying resistance mechanism of tyrosine kinase inhibitors (TKIs), ferroptosis-based combination therapy attracts more attentions from researches for taking advantage of possible synergy. Our preliminary finding displayed that low expression of ferritinophagy-related NCOA4 gene was correlated with decreased immune cells infiltration and impaired IFN-γ receptor signaling in ccRCC. So NCOA4 hold the expectation as a novel marker for identifying potentially eligible patients for the ferroptosis-induction treatment or its combination with immunotherapy.

## Data Availability

The data underlying this study are freely available from TCGA data portal (https://portal.gdc.cancer.gov/projects/TCGA-KIRC) and the GSE66271 and GSE53575 dataset (http://www.ncbi.nlm.nih.gov/geo/). The authors did not have special access privileges.

## Abbreviations

ARA70: Androgen receptor-associated protein 70
ccRCC: Clear cell renal cell carcinoma
FTH1: Ferritin heavy chain 1
GEO: Gene Expression Omnibus
HIF: Hypoxia inducible factor
HSCs: Hepatic stellate cells
GEPIA: Gene Expression Profiling Interactive Analysis
NCBI: National Center of Biotechnology Information
OS: Overall survival
PPI: Protein–protein interaction
RCC: Renal cell carcinoma
ROS: Reactive oxygen species
TCGA: The Cancer Genome Atlas
TIMER: The Tumor Immune Estimation Resource
VHL: Von Hippl-Lindau
